# Redefining Thermal Regimes to Design Reserves for Coral Reefs in the Face of Climate Change

**DOI:** 10.1371/journal.pone.0110634

**Published:** 2014-10-21

**Authors:** Iliana Chollett, Susana Enríquez, Peter J. Mumby

**Affiliations:** 1 Marine Spatial Ecology Lab, College of Life and Environmental Sciences, University of Exeter, Exeter, United Kingdom; 2 Marine Spatial Ecology Lab, School of Biological Sciences, Goddard Building, University of Queensland, Brisbane, Australia; 3 Instituto de Ciencias del Mar y Limnología, Universidad Nacional Autónoma de México, Puerto Morelos, México; Leibniz Center for Tropical Marine Ecology, Germany

## Abstract

Reef managers cannot fight global warming through mitigation at local scale, but they can use information on thermal patterns to plan for reserve networks that maximize the probability of persistence of their reef system. Here we assess previous methods for the design of reserves for climate change and present a new approach to prioritize areas for conservation that leverages the most desirable properties of previous approaches. The new method moves the science of reserve design for climate change a step forwards by: (1) recognizing the role of seasonal acclimation in increasing the limits of environmental tolerance of corals and ameliorating the bleaching response; (2) using the best proxy for acclimatization currently available; (3) including information from several bleaching events, which frequency is likely to increase in the future; (4) assessing relevant variability at country scales, where most management plans are carried out. We demonstrate the method in Honduras, where a reassessment of the marine spatial plan is in progress.

## Introduction

Global warming and ocean acidification represent an increasing threat to reef ecosystems [1]. Many studies have assessed likely responses of coral reef organisms to thermal stress and the expected changes in aragonite saturation state associated with ocean acidification (reviewed in [2], [3], [4]). However, research is still ongoing to understand the physiological mechanisms that explain thermally induced coral bleaching and responses to acidification, the magnitude of their impact on coral calcification, and the ecosystem-level consequences of these disturbances.

Stabilizing carbon emissions is the most critical action needed to address global climate change impacts, but this task is beyond the scope of on-the ground conservation managers. The environmental effects of global impacts can, however, be taken into account during the design of management interventions, such as marine reserve networks, by considering the differential vulnerability of reefs to the stressor(s) and selecting sites for protection that increase the probability of persistence of the whole network under a changing climate [5], [6], [7], [8].

Both bleaching and ocean acidification are associated with changes in oceanographic conditions: while mass bleaching is linked to acute increases in sea temperatures [9], severe reductions in coral calcification rates are expected from decreases in aragonite saturation state related to ocean acidification [10]. These variables, sea temperature and aragonite saturation state, can be mapped [11], [12], depicting a heterogeneous seascape which translates into spatially variable risks to environmental changes. To date, planning for ocean acidification is in its infancy [13], the response of organisms (e.g. decreased growth and calcification) is not as unequivocal as the impact of thermal stress and coral bleaching [13], [14], and the mapping of this threat is based on complex models that are still under development [11], [13], [15].

**Figure 1:**
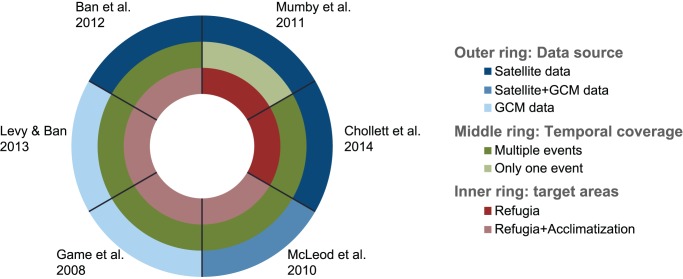
Comparison of methods available to prioritize areas for conservation in a climate change context. Contrasts in terms of the source of the data used (blue ring), the temporal coverage of the analyses (green ring) and the type of areas targeted for protection (red ring).

Spatially explicit information on patterns of sea surface temperature has been used to design reserve networks that consider the threat of bleaching (e.g. [5], [7]). Broadly, the methods differ in three ways: the type of data used as input for the analyses, their temporal coverage and the type of areas targeted for protection (Figure 1). Below we assess these three aspects and identify the strongest elements of all methods, which we then include when crafting an optimal approach to plan for marine reserve design in a climate change context.

So far reserve design methods have used satellite temperature data [7], [16], global climate models (GCM, [5], [17]) or a combination of both [18]. GCMs have the advantage of offering insight into future patterns, but have a coarse spatial resolution (hundreds of km), do not take into account processes at the reef scale, and are therefore too uncertain to be a foundation for conservation planning [19]. Satellite data, conversely, is better at capturing reef-scale variability, but is based on historical data, and therefore assumes that future incidence of bleaching events will occur under similar conditions as past incidences. Given that GCMs are unable to predict future bleaching events at a spatial scale relevant for management, the establishment of a method based on high-resolution historical data, which could be updated as new data becomes available, provides the most appropriate present-day option for managing reefs for climate change.

Currently planning approaches include data from a single or several bleaching events (Figure 1). Since the level of thermal stress can vary widely from one bleaching event to another and the spatial distribution of bleaching varies widely between events [16], [20], a robust approach to design reserve networks should include input data from multiple years and bleaching events.

Finally, existent methods also differ regarding the type of areas to prioritize. Most efforts target areas naturally least affected by thermal anomalies, assuming that climate change needs to be avoided for reefs to survive. Mumby et al. [7] presented the only framework that also considers the scope for acclimation and adaptation to increase the survival of corals under climate change. Although the magnitude of these processes is still uncertain, their relevance in an unstoppable climate change context could be considerable [21], [22], especially given the uncertain role of refugia in a changing climate [23] and therefore we suggest their inclusion in management plans.

Several environmental variables have been associated with higher rates of adaptation and/or acclimation to thermal stress, such as higher summer temperatures [7], [24] or higher temperature variability [25], [26], under the premise that organisms exposed to ‘stressful’ temperatures can develop ecological or physiological responses that will allow them to withstand acute thermal stress better. However, it has recently been recognized that one of the most relevant variables modulating bleaching occurrence is the rate of seasonal warming from spring to summer, because this variable can explain how well prepared the coral is at this critical time of the year [27], [28]. Corals that are subjected to rapid seasonal increases in temperature will experience faster losses in coral pigmentation, which makes corals more sensitive to light stress (a key component of bleaching), and therefore aggravates their response in the face of acute stress conditions [27], [28]. Despite its relevance for predicting the likelihood of bleaching, this new knowledge has not yet been incorporated in methods for prioritising reef areas for conservation.

Taking advantage of recent developments in the field, we propose an improved method for prioritizing areas for conservation that leverages the most desirable properties of previous methods and also incorporates the best proxies for acclimatization currently available. Specifically, the method combines freely available high-resolution satellite imagery, multiple bleaching events and target both areas naturally resistant to bleaching and potential areas of fast acclimation. We demonstrate the method in Honduras, western Caribbean, where a reassessment of the marine spatial plan is in progress.

## Methods

### Data set

Temperature was measured using satellite data provided by the Advanced Very High Resolution Radiometer (AVHRR) Pathfinder project v 5.2, freely available (http://pathfinder.nodc.noaa.gov). The 5.2 dataset is an updated version of the Pathfinder Version 5.0 and 5.1 collections described in Casey et al. [29].

The temperature dataset covers the period 1982–2010 and has a spatial resolution of 4×4 km. Only night data of quality 4 or higher was used [30]. Weekly averages were calculated from the daily data and data gaps were filled using temporal and spatial interpolation. First, any data gaps were identified and substituted by the average of the previous and following week for that particular pixel. Any remaining gaps were filled through spatial interpolation for the specific week using a spring metaphor, as described in Chollett and Mumby [23].

### Processing

**Figure 2:**
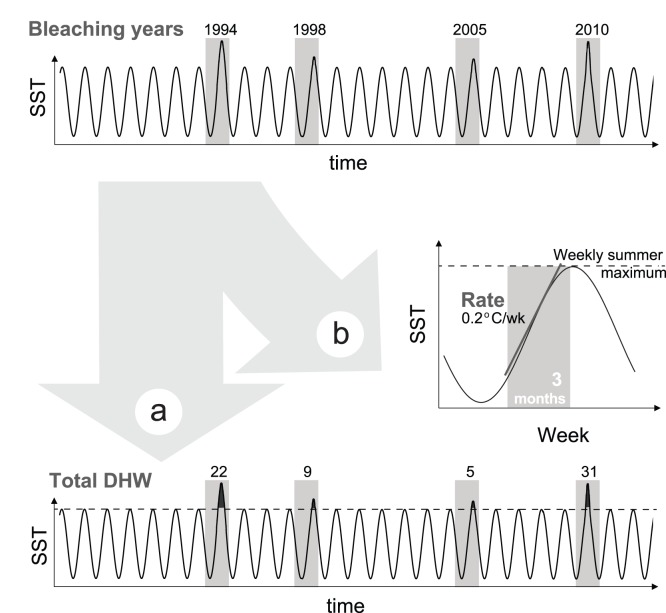
Temperature metrics extracted from each weekly time-series of sea surface temperature. (a) Total DHWs per bleaching year; (b) rate of increase in temperature.

From this weekly, gap-filled dataset, two metrics were derived: total amount of acute thermal stress per year, and average rate of increase in temperature from spring to summer (hereafter only ‘rate of increase in temperature’, Figure 2). Acute thermal stress was quantified as the total amount of Degree Heating Weeks (DHW, [31]) experienced in a particular year (Figure 2a). DHWs are a measure of accumulated thermal stress by summing temperatures 1°C above the usual summertime maximum over a 12 week period. Temperature increases above normal summer ambient have shown to induce bleaching [32], [33], and DHWs have been commonly related to the occurrence of bleaching and associated mortality [34], [35]. Finally, to calculate the rate of increase in temperature weekly climatological means using all data were computed, and the maximum of those selected as the summer maximum. Then the slope of the line of the three-month period prior to that date was calculated (Figure 2b).

Temperature metrics were extracted for reef areas within the Exclusive Economic Zone of Honduras. EEZ’s were extracted from the VLIZ Maritime Boundaries Geodatabase (http://www.marineregions.org). Data on location of coral reefs were collated by the World Resources Institute and includes information from the Millennium Coral Reef Mapping Project [36], the UNEP-WCMC coral reef map and other sources [37]. Data on temperature metrics for reef areas in the entire Caribbean are available at PANGAEA data repository (http://dx.doi.org/10.1594/PANGAEA.836153).

### Mapping thermal regimes using acute stress and rates of increase in temperature

Information on the acute thermal regime and rates of increase in temperature can be used to inform spatial variability in vulnerability to bleaching and prioritize areas for conservation anywhere. These two factors affect both the long and short-term acclimation of corals to thermal stress, and incorporate the best science available.

**Figure 3:**
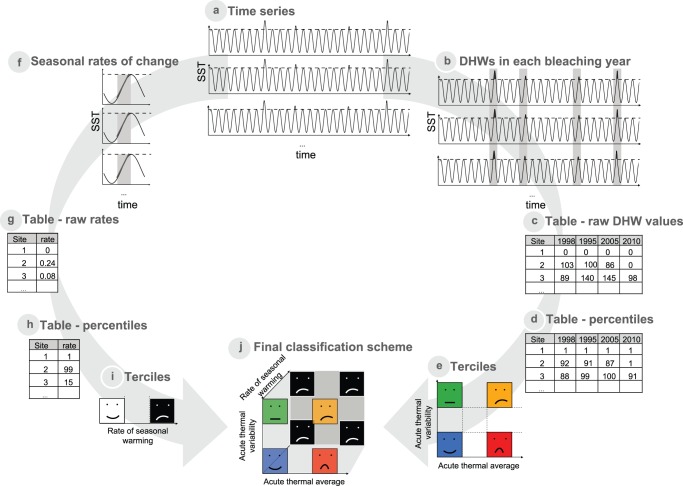
Diagram showing the overall methods used. (a) In each location, sea surface temperature time series are extracted and (b) the total amount of DHWs per bleaching event is calculated in each site. These values are (c) tabulated, (d) transformed into percentiles, and then (e) the average and standard deviation is calculated and plotted. This way the sites can be separated according to the intensity of acute thermal stress (weak or severe) and its variability (recurrent or not). Additionally, in each location, (f) the rate of change in temperature is quantified from the time series, (g) tabulated, (h) transformed into percentiles and (i) divided in terciles. (j) These two classifications are then incorporated into one final classification scheme.

This new method builds on information of all previous mass bleaching events (e.g. in the Caribbean 1995, 1998, 2005 and 2010: [35], [38], [39], [40]) and occurs in a two-step fashion, first incorporating data on how intense and frequent acute thermal stress events have been, and then adding information on the rate of increase in temperature in each area, which can modulate the expected outcome (Figure 3).

#### Acute stress

Acute temperature stress, the total amount of DHWs, is first quantified for each location and bleaching year (Figure 3b), the values are tabulated (Figure 3c) and transformed into percentiles so for each location we have 4 different percentile values, one for each mass bleaching event (Figure 3d). Raw DHWs are transformed into percentiles to rank locations and allow easier interpretation and comparisons among them. Subsequently the average and standard deviation of stress is calculated for each location. Note that areas with low standard deviation have either rarely experienced a thermal event or always experienced one. For example, site 3 in Figure 3 has been affected by thermal stress in every single event with high intensity (Figure 3c), which translate into high percentiles (88^th^–100^th^ percentile), and therefore high average (95) and low variability (6).

Average and variability measures are then divided independently into three groups (terciles) and locations at the extremes of each stress measure (i.e. upper and lower thirds) are used to generate four contrasting temperature stress regimes (Figure 3e). Using this information, reefs can be classified into four categories based on their exposure to high temperature: Blue, Green, Yellow and Red. (Blue) areas that have received weak acute thermal stress during all bleaching events and constitute a refugia against thermal disturbances; (Green) areas that have been subjected to generally weak, albeit variable, acute stress, providing some potential for adaptation; (Yellow) areas that have suffered severe but variable acute stress, should do worse than (Green), and still have potential for adaptation but with differential fitness among species and therefore lower diversity; and (Red) areas that have suffered severe acute stress in a constant manner, which will likely experience higher reductions in coral fitness and fare worst by climate change.

#### Rates of increase in temperature

The other element to take into consideration is the average rate of change in temperature between spring and summer for each location. For all years, weekly average temperatures are calculated, and the maximum of those selected as summer maximum. Then the slope of the line of the three month period prior to that date is calculated (Figure 3f). Rates are tabulated (Figure 3 g), transformed into percentiles (Figure 3 h) and then divided into terciles (Figure 3i), allowing contrasting conditions to be identified. This information can be integrated with data on acute thermal stress events (Figure 3j) such that high rates of change in temperatures aggravate the prognosis of the reef and its likely fate after bleaching. The physiological rationale of including rates of increase in temperature as a proxy for short-term acclimation can be found in the file S1.

## Results

**Figure 4:**
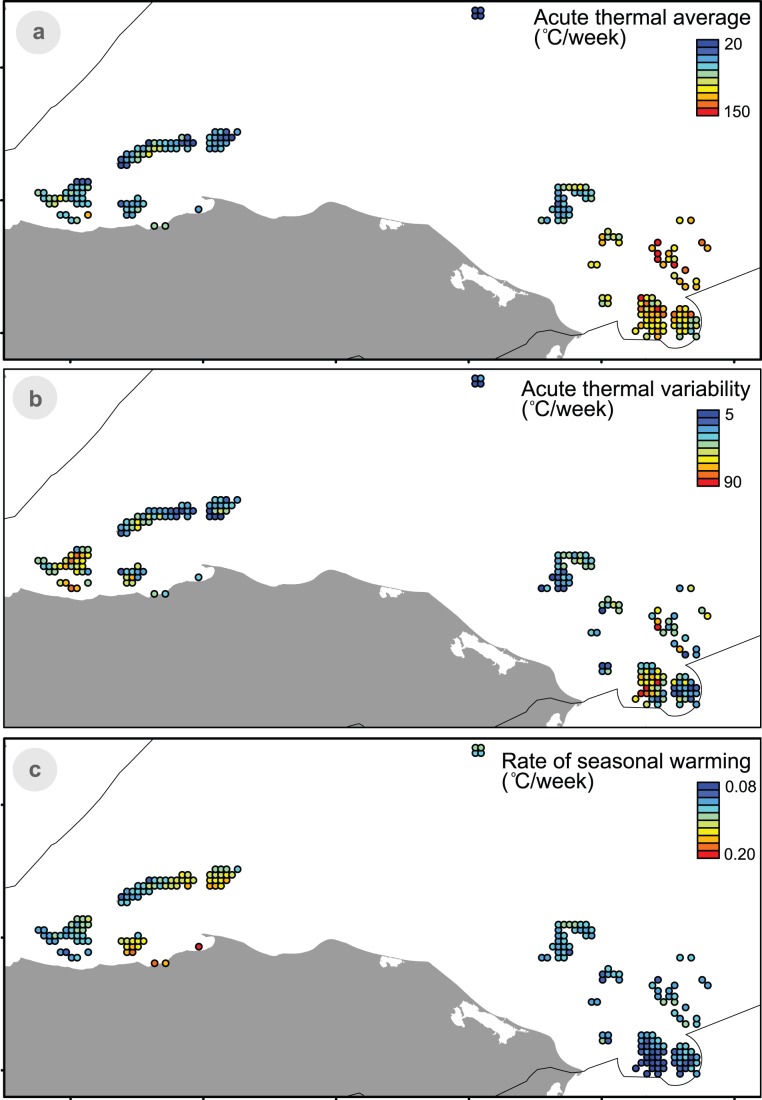
Temperature patterns in reef areas inside the Honduran EEZ. (a) Average acute thermal stress; (b) variability in acute thermal stress; and (c) rate of warming.

**Figure 5:**
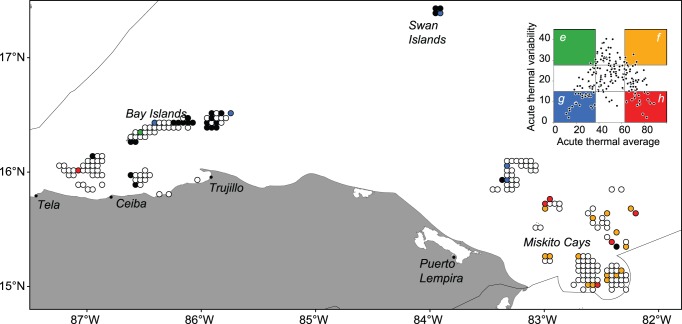
Spatial arrangement of thermal regimes in Honduras. Classification of extreme categories following the colour scheme in Figure 3j with areas with high rate of warming are depicted in black. Unclassified areas are depicted in white. Inset shows average vs. standard deviation percentiles of thermal anomalies in four Caribbean-wide bleaching events (1995, 1998, 2005 and 2008) in reef habitats in Honduras.

Honduran waters are largely heterogeneous in terms of acute thermal stress and rate of increase in temperature (Figure 4). Acute stress is more intense in the mid and southern Miskito Cays (Figure 4a), and more variable in the south-west Miskito Cays and the western side of the Bay Islands (Figure 4b). In contrast, higher rates of seasonal warming are found in the eastern portion of the Bay Islands and areas along the coast south of the Islands. As a result, distinct thermal regimes are well distributed across Honduras (Figure 5), with areas of low overall acute thermal stress (Blue) mostly concentrated in the north of the region along the Bay and Swan Islands and northern Miskito Cays, and areas of high acute thermal stress (regimes Yellow and Red) in the central and southern section of the Miskito Cays.

Interestingly, areas of fast rates of summer warming mostly (88% of the time) occur in Blue refugia regimes, rarely in Yellow (8%) or Green (4%) regimes, and never in Red regimes. The results for Honduras suggest that a network of Marine Protected Areas that accounts for climate change impacts and scope for adaptation should include representation in the southern Miskito Cays and the Bay Islands (particularly in the north of the middle island –Roatan- and eastern island -Guanaja), probably also incorporating reefs in the northern Miskito Cays and the Swan Islands to work as stepping stones if relevant for connectivity within the area.

## Discussion

Freely available sea surface temperature data can be used to plan for global warming by integrating information from multiple bleaching events and considering the capability of corals to adapt to the changes while using the best science available. The method introduced here has two main advantages over alternative approaches for reserve design in a climate change context. First, unlike most methods (Figure 1), it takes into account the ability of corals to adjust to climatic changes by using the latest advances in bleaching science available. Secondly, it incorporates the impacts of multiple bleaching events (lacking in [7]), and therefore includes natural temporal (as well as spatial) variability in the stress associated with bleaching.

Our temperature regimes coincide with well-defined oceanographic regions. Refuge areas (Blue regime) encompass environmental conditions that afford natural resistance to bleaching, and as a consequence are relatively unaffected by thermal anomalies [41]. Refugia include areas of moderate depths, regions exposed to vigorous circulation or high wave exposure, upwelling and high-latitude areas [41]. The protection afforded by areas naturally resistant to bleaching has long been identified as an important component of MPA networks in a changing climate [5], [6], [16], [42]. If a conventional approach to reserve design has to be taken, then reserves should be located in these areas. However, we recommend following a more unconventional approach, and also include areas that have experienced stress in the past, which might prepare corals better for future temperature anomalies. This approach recognises the potential of corals to adapt and acclimatise, and also takes into account the fact that many areas allegedly resistant to bleaching are incapable of avoiding thermal stress in a significant and consistent manner through time, at least in the Caribbean [23].

Reefs where thermal stress is intense and recurrent (Red regime), will experience high coral mortality and less opportunity to recover, acclimatise or adapt [33]. Longer time periods between disturbances, and therefore high standard deviations in acute stress, are more likely to lead to higher recovery and reef persistence over time. These regimes (Yellow and Green) are key if long-term acclimation of corals is to be considered in marine reserve design. A large number of empirical studies suggest that corals that have been previously exposed to moderate levels of thermal stress have greater resistance to future events [43], [44], [45]. These changes might occur due to community shifts towards dominance of coral-tolerant taxa [44], or acclimation due to changes in symbiont community composition through switching or shuffling [46] or changes in the host [47], [48]. Under intense, albeit variable bleaching (Yellow regime) the potential for acclimation/adaptation will depend both on the survival of corals after bleaching and the time frames required for acquiring genetic adaptation and phenotypic acclimation, which are uncertain.

Selecting a variety of temperature regimes might increase the likelihood that some reefs will survive future bleaching [6]. To maximize chances of success, managers might focus protection on areas that are predicted to cope better with climate change, which have been less impacted by thermal stress at all times (Blue regime). If feasible, including areas that offer the greatest potential for acclimation (areas with high variability in incidence of thermal stress such as the Green or Yellow regimes) could serve as a bet-hedging strategy. Areas subjected to severe, constant stress (Red regime) should be avoided, as well as areas with rapid increases of temperature from spring to summer. This information should be factored in with other relevant layers (e.g. connectivity, habitat maps, maps of current uses and threats) to prioritize conservation actions. See Magris et al. [49] for a recent review on how to incorporate thermal regimes and connectivity patterns into marine spatial planning, or Zanakis et al. [50] for methods to incorporate multiple attributes into decision making and spatial planning.

Satellites measure the first few millimeters of the ocean surface, literally the “skin” of the ocean. The water column is dynamic and satellite temperature data do not mirrors entirely the temperature patterns on the seabed, where corals are located, missing some small-scale variability and extremes [20], [51]. In spite of this limitation, satellites are unique at allowing the assessment of large areas simultaneously, and a key tool for marine spatial planning. Additionally, another constraint of the method described here is that is based on historical data, which has some limitations for use in planning for climate change when assessing rare (in a climatological context), extreme disturbances such as bleaching events, given that implicitly assumes that future incidence of bleaching events will be similar than past incidence. The method, however, could be refined by adding new information as it becomes available (i.e., as additional bleaching events occur) and be incorporated into an adaptive planning process. As we mentioned before, Global Circulation Model outputs, given their coarse resolution, are not appropriate for site selection of MPAs [19]. Techniques are available to obtain local weather patterns from GCM results, based either on statistical downscaling (by using historical information: [52]) or dynamical downscaling (by integrating global and regional models). Statistical downscaling assumes that the relationship established between the modeled data and the historical data for the present day climate will hold in the future, and that there will be no systematic changes in regional forcing at scales not considered by the global model [53], which makes them unrealistic for the assessment of future changes in coastal areas. Dynamical downscaling would be the best alternative to plan for climate change at a spatial resolution relevant for reefs; however, this method is computationally intensive and as a consequence it has never been used for marine spatial prioritization to date.

Finally, we anticipate that further research when planning for coral bleaching under a changing climate will move away from looking merely at sea temperature data to look into new synoptic satellite products that incorporate both light and temperature stress, which are currently under development [54]. While mass-bleaching events have been associated with anomalous increases in sea surface temperature, other environmental factors can also contribute to modulate the bleaching response. Among them, light plays a key role in the determination of the severity of the temperature impact, as it exacerbates its effect increasing the levels of oxidative stress accumulated in the coral tissue [27], [28], [55], and determining the levels of pigmentation in the coral and therefore the response to thermal stress [56], [57]. These new synoptic satellite products that incorporate both light and temperature constitute the next frontier when planning for coral bleaching under different global warming scenarios.

## Supporting Information

File S1 Why the rate of seasonal warming is relevant at modulating bleaching incidence – inside knowledge.
(DOCX)
