# Supplementary material for: Redefining Thermal Regimes to Design Reserves for Coral Reefs in the Face of Climate Change
Source: PLoS One. 2014 Oct 21;9(10):e110634. doi: 10.1371/journal.pone.0110634 (PMC4204933; doi:10.1371/journal.pone.0110634)
Supplement: File S1 — Why the rate of seasonal warming is relevant at modulating bleaching incidence – inside knowledge. (DOCX) [file pone.0110634.s001.docx]

**Supplementary material S1**

**Why the rate of seasonal warming is relevant at modulating bleaching incidence – inside knowledge**

The rate of seasonal warming from spring to summer plays a central role in the determination of the summer phenotype, therefore modulating bleaching occurrence. Seasonal increases in light and temperature trigger the natural summer reduction in coral pigmentation [1,2]. The speed of these changes will be key in determining the summer pigmentation of corals [1,2,3]. Corals that are subjected to rapid increases in temperature from spring to summer will experience faster losses in coral pigmentation and symbiont content, developing a more fragile phenotype, which will tend to fare worse in the face of acute stress conditions.

With less coral pigmentation the response to acute thermal stress is aggravated because of increased light stress and cumulative photodamage in pale corals [4,5,6,7,8]. The damage occurs either simply because the remaining symbionts in less pigmented corals absorb more light [8], or because the lack of pigments enhance the internal light field *in hospite* in comparison with more pigmented organisms [5] which induces low pigmented corals to experience larger photodamage under similar levels of thermal stress. Contrarily, higher coral pigmentation in summer allows reducing photodamage accumulation in the corals under similar levels of thermal stress because of pigment self-shading within the host tissue, which minimizes light damage.

Corals also adjust to seasonal increases in temperatures by reducing respiration through decreases in pigmentation and biomass [2] and by inducing the gene expression of the summer phenotype [9]. This natural process may affect the fitness of the organism (growth, calcification, reproduction) which is trying to keep up with the environmental changes. If increases in temperature occur rapidly and losses of coral pigmentation are too large, the capacity of corals to adjust to further increases in temperature will be compromised and bleaching will be more likely.

Yet, any local condition (such as steady increases in temperature from spring to summer) which provides enough time for corals to fully develop the summer phenotype and acclimate to summer temperatures maintaining higher pigmentation and sufficient number of symbiotic cells will strongly reduce the severity of the light stress to a given bleaching event.

**References**

1. Fagoonee I, Wilson HB, Hassell MP, Turner JR (1999) The dynamics of zooxanthellae populations: a long-term study in the field. Science 283: 843-845.

2. Fitt WK, McFarland FK, Warner ME, Chilcoat GC (2000) Seasonal patterns of tissue biomass and densities of symbiotic dinoflagellates in reef corals and relation to coral bleaching. Limnology and Oceanography 45: 677-685.

3. Brown BE, Dunne RP, Ambarsari I, Tissier MDAL, Satapoomin U (1999) Seasonal fluctuations in environmental factors and variations in symbiotic algae and chlorophyll pigments in four Indo-Pacific coral species. Marine Ecology Progress Series 191: 53-69.

4. Warner ME, Fitt WK, Schmidt GW (1999) Damage to photosystem II in symbiotic dinoflagellates: a determinant of coral bleaching. Proceedings of the National Academy of Sciences 96: 8007-8012.

5. Terán E, Méndez ER, Enríquez S, Iglesias-Prieto R (2010) Multiple light scattering and absorption in reef-building corals. Applied Optics 49: 5032-5042.

6. Jones RJ, Hoegh-Guldberg O, Larkum AWD, Schreiber U (1998) Temperature-induced bleaching of corals begins with impairment of the CO2 fixation mechanism in zooxanthellae. Plant, Cell & Environment 21: 1219-1230.

7. Iglesias-Prieto R (1997) Temperature-dependent inactivation of photosystem II in symbiotic dinoflagellates Proceedings of the 8th International Coral Reef Symposium. Panama City, Panama. pp. 1313–1318.

8. Enríquez S, Mendez ER, Iglesias-Prieto R (2005) Multiple scattering on coral skeletons enhances light absorption by symbiotic algae. Limnology and Oceanography 50: 1025-1032.

9. Bellantuono AJ, Granados-Cifuentes C, Miller DJ, Hoegh-Guldberg O, Rodriguez-Lanetty M (2012) Coral thermal tolerance: tuning gene expression to resist thermal stress. PLoS ONE 7: e50685.
